# Iatrogenic Liver Disease

**Published:** 1962-04

**Authors:** A. E. Read

**Affiliations:** Senior Lecturer, Department of Medicine, University of Bristol


### IATROGENIC LIVER DISEASE

BY

A. E. READ, M.D., M.R.C.P.

*Senior Lecturer, Department of Medicine, University of Bristol*

> Physicians of all men are most happy; what good success soever they have, the world proclaimeth and what faults they commit, the earth covereth.
>
> FRANCIS QUARLES
> 1592–1644.

The diseases which the physician produces are called iatrogenic (iatros—physician) but this, of course, does not mean that the surgeon is particularly innocent in this respect or less to blame than his medical colleagues. Liver disease seems to have perhaps more iatrogenic "possibilities" than disorders of other organs, and in order to discuss them briefly it is best to consider iatrogenic disorders as of two varieties. Firstly iatrogenic disease may be a direct result of therapy or investigation, and secondly it may be considered as resulting from faulty diagnosis or inappropriate treatment. Furthermore in respect to the liver one can consider iatrogenic lesions in relation to the features of liver cell failure, namely jaundice, fluid retention, neuropsychiatric complications, and haemorrhage associated with portal hypertension. A further group is associated with abnormalities of hepatic structure, and these may or may not be accompanied by evidence of disordered liver cell function.

### *Iatrogenic Jaundice*

This is largely synonymous with drug jaundice. Drugs can produce jaundice in two ways. Firstly they can produce haemolysis and so overload a normal liver. Secondly they may produce actual liver disease, either by obstructing the intrahepatic biliary canaliculi or by injuring the liver cells. In the second group obstructive jaundice is produced by drugs such as methyl testosterone, chlorothiazide, thiouracil, sulphonomides, arsphenamine, chlorpropamide and chlorpromazine. Chlorpromazine is perhaps the best known of these drugs, because of its recent widespread use, and it causes jaundice in a small percentage (about 1 per cent) of patients to whom it is administered. It is usually stated that chlorpromazine jaundice is due to allergy on the part of the patient, but not all the drugs in this group act in this way. Although obstructive jaundice resulting from drugs may be persistent and severe, and may continue on rare occasions for many months long after the drugs have been withdrawn, there is little evidence suggesting that irreversible damage results to the liver and "primary" biliary cirrhosis is probably not the end result of this type of reaction.

Drugs which may injure liver cells and produce jaundice are far more numerous. In a recent survey there were thought to be about 150 drugs which can act in this way including anti-epileptic, anti-tuberculous and anti-rheumatic agents and others. The most recently recognized are the tranquillizer drugs particularly the amine oxidase inhibitors such as iproniazid (Marsilid). With regard to Marsilid it has been shown that the clinical picture is practically indistinguishable from acute infective hepatitis. Although it is not necessary or indeed possible to remember all the drugs which can cause jaundice it *is* necessary to remember that drugs *can* cause jaundice. An essential part of the history of any patient with jaundice is to enquire about drugs being taken.

The patient's relatives, family doctor, and pharmacist must all be questioned in suspicious cases and if this is done unnecessary and often dangerous surgical intervention can be avoided. In respect of the drugs producing liver cell jaundice it must be remembered that this is a dangerous condition and fatalities are not rare.

The surgeon is, of course, concerned with jaundice; he occasionally may play a part in its production as when the common bile duct is injured and stricture formation results. The end result of the sepsis and biliary retention which results from this is the production of a secondary biliary cirrhosis with the features both of liver cell failure and portal hypertension as well as these of chronic obstructive jaundice. One point of importance which has arisen from the concept of biliary obstruction due to lesions inside the liver at the level of the biliary canaliculus, is that this type of jaundice is of course not amenable to surgical therapy. Some types of obstructive jaundice are indeed "medical" disorders. Intrahepatic biliary obstruction forms a small but important percentage of all cases of obstructive jaundice, best recognized if possible before laparotomy.

One cannot discuss iatrogenic jaundice without mentioning the well-known fact that serum hepatitis is a pure example of this type of disorder. With the rare exception of the lesion produced by the tattoo artist, most other examples of this disease can be blamed on the doctor, dentist, or nurse. The other virus affection of the liver (infective hepatitis) is on the other hand not usually iatrogenic although it can be, as the virus circulates in the blood stream during the active phase of the disease and contaminated needles can, therefore, carry it.

### *Fluid Retention*

Ascites occuring during the course of cirrhosis usually denotes a poor prognosis. There is a tendency for the surgeon on some occasions to perform a laparotomy to find the cause of ascites. This is quite in order if it is certain that cirrhosis is not the cause. The surgeon's knife is a good test of liver function and if cirrhosis is the cause of ascites the diagnosis can be brought home very vividly when the patient goes into coma following his or her operation. Hepatic coma is not uncommon following surgical drainage of ascites or following paracentesis and, therefore, ascites should if possible be treated with diuretics and sodium restriction. A very important additional reason for insisting upon medical rather than "surgical" treatment of ascites is that protein is not drained from the body by diuretics. Hypoproteinaemia is always present in cirrhosis with ascites. Paracentesis makes it more severe and encourages fluid retention at an accelerated rate. The importance of medical treatment was apparent to Celsus (20 B.C.) who before advising paracentesis advised more conservative therapy. In fact he particularly favoured applications of bruised figs and honey to the abdomen, and perhaps therefore it is not surprising that he gained more success from paracentesis.

It is not unusual, I am afraid, for laparotomy to be used as a way of diagnosing cirrhosis. Many a cirrhotic patient wears an abdominal scar but should not receive one until the surgeon has at least looked at the liver function tests. One must remember however, that many physicians have treated patients with cirrhosis for "cardiac failure" simply because there was ankle oedema with cirrhosis, or because ascites when present was sufficient to produce jugular venous engorgement.

The other major iatrogenic lesion following the treatment of patients with ascites is peritoneal infection. Although the cirrhotic patient is susceptible to *B.coli* septicaemia, perhaps because blood in abnormal anastomotic vessels bypasses the liver where bacteria are destroyed, there is also a possibility of infection being introduced from without or even from a small perforation of the gut by the trocar.

Abdominal infection in the cirrhotic patient is not manifest with dramatic physical signs. The patient may complain of abdominal discomfort and there is a pyrexia.

The patient usually lapses into hepatic coma and certainly it is very important to think of infection in the abdominal cavity as a cause of coma in a patient with cirrhosis and ascites particularly if there has been a recent paracentesis.

*Bleeding from Portal Hypertension*

Oesophageal varices frequently rupture, and the cause for this is unknown in many cases. Patients with terminal liver failure often bleed from varices and it is assumed in these cases that bleeding is as much a manifestation of liver failure as it is of portal hypertension. Bleeding can rarely follow splenic venography presumably because of increased pressure from the injection of contrast material. Bleeding from varices is not uncommon following paracentesis abdominis; the reasons for this are not well understood but the danger should be remembered.

One other important cause of iatrogenic haemorrhage may be related to the admission and immobilisation of patients with cirrhosis and ascites in hospital beds. Presumably the enforced recumbent position coupled with the increased intra-abdominal pressure due to ascites is likely to encourage oesophageal reflux. This may well be enough to cause erosion of oesophageal varices, for certainly it is not unusual for cirrhotic patients to bleed for the first time in hospital. This I found to be so in one series of cirrhotics in whom about 25 per cent experienced their first bleed from varices in hospital. Such an event is often fatal particularly in patients with complicating ascites and it is perhaps a factor which should be born in mind before deciding whether the ascitic patient needs hospital admission.

Portal hypertension is often associated with dilatation of the azygos venous system, as this system of veins carries the flow of many of the collateral vessels which develop because of portal hypertension. It may indeed even produce abnormal X-ray shadows in the chest and it has even led to thoracotomy in a patient with portal hypertension for supposed neoplasm in the chest. This is perhaps an extreme example of an iatrogenic lesion, in this case due to a failure to appreciate the significance of chest shadows caused by portal hypertension.

Although bleeding may occasionally be caused by iatrogenic factors, it must be remembered that its treatment may be similarly complicated. Hepatic coma and precoma may result from porto-caval anastamosis but this will be discussed briefly later. The Sengstaken-Blakemore compression tube is one apparatus used in the treatment of bleeding oesophageal varices and it is one way in which iatrogenic disorders may arise. Ulceration of the larynx and oesophago-gastric junction, inhalation pneumonia and laryngeal obstruction because of migration of the apparatus through the cardiac sphincter are not uncommon if the apparatus is used for longer than 24 hours at a time or if traction is used with it. Despite the hazards which sometimes attend the use of this apparatus it may on occasions be a valuable therapeutic weapon—even life saving.

*Hepatic Coma*

It has been customary to regard hepatic coma as a disorder in which cerebral function is changed because of the presence in the cerebral blood of some toxic substance or substances related to the bacterial decomposition of protein in the intestine. This set of circumstances presumably arises because of failure of liver cells to detoxicate these products, and because collateral vessels which arise because of accompanying portal hypertension carry these products directly to the systemic circulation. There is a tendency nowadays to regard hepatic coma as a condition arising from a wide variety of metabolic stimuli. These produce the same clinical picture and some of them are unrelated to any factor which could be associated with alimentary protein metabolism. In other words the response of the patient with liver disease to a wide variety of metabolic stimuli may be manifest clinically as hepatic coma.

Amongst various iatrogenic factors concerned in this respect are some sedative drugs. Morphine has been said to be dangerous—because of presumed delay in excretion—in patients with liver disease. We were able to demonstrate the profound effect of even small doses of morphine on the E.E.G., slowing of the record occurring just as it does in spontaneous hepatic coma. So impressed were we by the brisk reaction of patients with hepatic cirrhosis and a tendency towards neuropsychiatric complications (past history of confusion, evidence on examination of mental confusion, hepatic foetor and tremor, etc.) that we even used it with the E.E.G. to show the liability of a patient to develop hepatic coma—a not unimportant question in considering for example the risk attached to operations such as portocaval anastamosis.

Diuretics are also liable to produce neuropsychiatric complications in patients with cirrhosis and fluid retention. Ammonium salts such as ammonium chloride are toxic, probably because of the ammonia content. Diamox (acetazolamide) has also been shown to be toxic and produces neuropsychiatric complications probably because it affects ammonia metabolism in the peripheral tissues and in the renal tubule. The first really effective oral diuretic, chlorothiazide, has been shown to produce similar trouble, but here the cause seems to be related to potassium loss induced by the diuretic. This leads to hypokalaemia and this or the extracellular alkalosis it produces somehow brings about neuropsychiatric deterioration. It is interesting to note that potassium deficiency induced in other ways can also lead to neuropsychiatric deterioration in patients with cirrhosis. Aldactone when used in conjunction with thiazide derivatives in the control of fluid retention in cirrhotics reduces urinary potassium loss and makes these diuretics less dangerous from the point of view of neurological complications.

Hepatic precoma and coma are also seen in patients who have been subject to portocaval anastamosis. This may thus be considered to be an iatrogenic lesion despite the necessity for performing such operations to treat bleeding from oesophageal varices. It is said that hepatic coma or precoma is seen in 10–20 per cent of patients following this operation. It is also important to remember however that the more carefully one looks for evidence of neuropsychiatric complications following porto-caval anastamosis, with sensitive clinical and E.E.G. techniques, the more commonly are they found, and the true figure even in carefully selected groups of patients may be in excess of this.

### *Iatrogenic "Structural" Disorders of the Liver*

The liver can develop cellular changes due to various iatrogenic factors and some of these may be manifest without prominent clinical features.

Fatty changes in the liver may be severe in patients who have had intestinal resections and similar state of affairs develops in other types of malabsorption syndrome. Failure to treat patients with idiopathic steatorrhoea adequately may lead to progressive liver disease, as there are still many clinicians and pathologists who feel that fatty infiltration can progress to cirrhosis. Certainly amongst patients with idiopathic steatorrhoea there is more than the usual incidence of cirrhosis, and perhaps iatrogenic factors may occasionally be responsible for it. Cortisone also produces fatty infiltration of the liver, and if it is used in patients with liver cell disease the histological changes produced may be confusing unless the possibility is borne in mind.

Iron deposits in the liver cell and the Küpffer cell occur in haemachromatosis, both primary due to excessive absorption of iron and secondary to the administration of iron compounds by mouth and parenterally. This can, therefore, be an important iatrogenic disorder as it is not uncommon to see patients with refractory anaemia who have been treated with iron compounds for many years. The refractory sideroblastic anaemias though rare are particularly prone to develop this syndrome because they already have excessive iron stores and iron therapy accelerates the deposit of tissue iron.

Other drugs and chemicals are known to produce histological changes in the liver, and mention may be made of the granulomatous reaction to beryllium, the foam cells resulting from the presence of polyvinyl pyrollidene (PVP) in liver cells, and the occasional occurrence of hepatic vein fibrosis following urethane.

This account must of necessity be incomplete but it is hoped that the facts that it contains will not alarm the clinician who deals with patients with liver disease. Although it is unfortunately true that the patient suffers because of iatrogenic disease, in respect of iatrogenic liver disease it must be said that through it much has been learnt regarding the nature of the other diseases of the liver and their treatment which has been of benefit to patient and clinician.

